# Skin cancer classification leveraging multi-directional compact convolutional neural network ensembles and gabor wavelets

**DOI:** 10.1038/s41598-024-69954-8

**Published:** 2024-09-04

**Authors:** Omneya Attallah

**Affiliations:** 1 Department of Electronics and Communications Engineering, College of Engineering and Technology, Arab Academy for Science, Technology and Maritime Transport, Alexandria, 21937 Egypt; 2 Wearables, Biosensing, and Biosignal Processing Laboratory, Arab Academy for Science, Technology, and Maritime Transport, Alexandria, 21937 Egypt

**Keywords:** Skin cancer diagnosis, Dermoscopic imaging, Deep learning, Convolutional neural networks, Gabor wavelets, Feature fusion, Feature selection, Biomedical engineering, Diagnosis, Computer science

## Abstract

Skin cancer (SC) is an important medical condition that necessitates prompt identification to ensure timely treatment. Although visual evaluation by dermatologists is considered the most reliable method, its efficacy is subjective and laborious. Deep learning-based computer-aided diagnostic (CAD) platforms have become valuable tools for supporting dermatologists. Nevertheless, current CAD tools frequently depend on Convolutional Neural Networks (CNNs) with huge amounts of deep layers and hyperparameters, single CNN model methodologies, large feature space, and exclusively utilise spatial image information, which restricts their effectiveness. This study presents SCaLiNG, an innovative CAD tool specifically developed to address and surpass these constraints. SCaLiNG leverages a collection of three compact CNNs and Gabor Wavelets (GW) to acquire a comprehensive feature vector consisting of spatial–textural–frequency attributes. SCaLiNG gathers a wide range of image details by breaking down these photos into multiple directional sub-bands using GW, and then learning several CNNs using those sub-bands and the original picture. SCaLiNG also combines attributes taken from various CNNs trained with the actual images and subbands derived from GW. This fusion process correspondingly improves diagnostic accuracy due to the thorough representation of attributes. Furthermore, SCaLiNG applies a feature selection approach which further enhances the model’s performance by choosing the most distinguishing features. Experimental findings indicate that SCaLiNG maintains a classification accuracy of 0.9170 in categorising SC subcategories, surpassing conventional single-CNN models. The outstanding performance of SCaLiNG underlines its ability to aid dermatologists in swiftly and precisely recognising and classifying SC, thereby enhancing patient outcomes**.**

## Introduction

Skin cancer (SC) is a common and serious disease caused by the uncontrollable proliferation of abnormal skin cells^1^. It is now a significant public health issue due to increasing rates of occurrence worldwide^2^. From 2008 to 2018, there was a 53% increase in the overall number of melanoma cases caused by excessive exposition to UV rays^3^. SC is categorised into various subtypes, with the most prevalent being basal cell carcinoma, squamous cell carcinoma, and the most severe form, melanoma^4^. Melanoma of the skin boasts a relatively high 5-year survival rate of 93.5%^5^. Identifying skin melanoma early leads to a 99.6% chance of surviving for 5 years^5^. Thus, timely identification and medical intervention greatly enhance the chances of survival, especially for melanoma^6^. Conventionally, the diagnosis of SC is mainly based on visual examination by dermatologists using dermoscopic images and occasionally requires biopsies for verification. There are still inherent drawbacks associated with manual diagnosis^7^. Subjective evaluations may result in inconsistencies, especially in situations involving subtle malignant characteristics or atypical manifestations because of complexities, noise, intensity variations, and similarities among lesions. Besides, dermoscopic examinations encounter challenges in detecting and categorising early-stage malignancies because of their tiny dimensions and subtle differences in size, form, texture, and colour on various skin surfaces. Furthermore, the scarcity of dermatologists in certain areas could hinder visual detection and lead to delayed diagnosis^8^.

To address these obstacles, computer-aided diagnosis (CAD) systems are surfacing as a potentially beneficial tool for physicians. Deep learning (DL), specifically convolutional neural networks (CNN), has significantly transformed the field of medical image analysis^9–11^. CNNs have a remarkable ability to derive complex features from images.^12^. DL techniques have also been extensively used to support the recognition of various diseases such as breast cancer^13,14^, cervical cancer^15–17^, lung diseases^18–20^, blood diseases^21^, heart diseases^22^, brain tumours^23,24^, and eye diseases^25,26^. The use of CAD systems, especially those powered by DL, offers a crucial avenue for early detection and improved diagnostic accuracy in SC^27^. Examining the literature revealed that although there are multiple diagnostic and classification methods for SC, there are still several gaps that need to be addressed. The factors involved are the detailed configurations, the higher complexities of the models used in specific studies, and the reduced level of diagnostic accuracy. Many studies used deep features extracted from large CNNs with many deep layers and huge parameters. Most previous CAD systems rely on separate CNNs for classification. However, combining more deep features from different CNNs with diverse structures could enhance diagnostic accuracy, as proposed by Karthik et al.,^28^ Nagaraj and Subhashini,^29^ Attallah^30^. Primarily, they used skin images as direct inputs to CNN models and relied solely on spatial information to predict the types of skin lesions. However, employing multi-resolution analysis such as Gabor Wavelets (GW) to decompose images and produce various subbands of images could boost the performance of CNNs. GW analysis can offer useful supplementary knowledge for CNNs in the context of SC diagnosis^26^. GW is proficient in isolating specific features from images that are localised, multi-scale, and orientation-specific^31^. This corresponds effectively to the visual attributes of skin lesions, in which texture, patterns, and edges at different scales are crucial diagnostic indicators^32^. CNNs can obtain a more detailed representation of skin lesions when using GW by breaking down the images into various texture, frequency, and orientation components (Gabor-filtered subbands). This may result in better performance, especially in situations involving subtle or intricate features of the lesion^33^. This is because, the texture and frequency information obtained by GW enhances the feature representation acquired by the CNN, potentially enhancing its diagnostic accuracy^33^.

In this study, a novel CAD based on three lightweight CNNs of distinct architectures and GW is proposed to diagnose SC called “SCaLiNG” which stands for skin cancer classification leveraging compact networks and Gabor wavelets. The proposed CAD involves breaking down input images into four directional sub-bands using GW. This approach offers multi-directional decomposition, with each sub-band producing distinct directional information in its corresponding channel. Five parallel CNNs having similar architectures are fed with these four subband photos and the original photos. Deep features are extracted for the five CNNs and combined. Each channel in a CNN generates learning specific to that direction, which can be considered independent from learning in other directions. Therefore, integrating the deep features extracted from these channels enhances the overall classification accuracy. Furthermore, SCaLiNG CAD merges deep spatial-textural-frequency features of the three lightweight CNNs benefiting from their unique architectures, thus improving performance. In addition, SCaLiNG CAD employs feature selection (FS) to select deep features that impact diagnostic accuracy.

The primary contributions and novelty of this article can be summarised as follows :

- Instead of directly using the input images to train the CNN, SCaLiNG CAD involves breaking down input images into four directional sub-bands and using them, along with the original image, as inputs for five parallel CNNs.
- Employ lightweight CNNs instead of CNNs with a huge number of parameters and deep layers
- Using a variety of CNNs with distinct structures, instead of traditional CAD models for SC diagnosis that depend on a single CNN.
- Combining deep features that are obtained from CNNs trained with different subbands of GW produces distinct directional, texture, and frequency information rather than using only spatial data, which is the case in existing CADs.
- In order to streamline and reduce the classification model complexity of the training process, a FS approach is employed to determine a more concise and significant subset of attributes from the merged deep features.
- SCaLiNG CAD simplifies the complexity of the CAD model by not requiring segmentation or enhancement operations.

This paper is structured as follows. Section "Literature review" reviews existing research on using DL for SC diagnosis. Section "Methods and materials" details the methodology employed in this study. The experimental setup is described in Section "Experimental setting". Section "Results" presents the results of a comprehensive assessment and analysis, followed by Section "Discussions" which discusses the key findings and limitations of SCaLiNG CAD, and suggests potential areas for future research. Finally, Section "Conclusion" concludes the paper.

## Literature review

The literature on SC diagnosis shows that different DL models were used to recognise multiple subclasses of SC including melanoma, melanocytic nevus, basal cell carcinoma, actinic keratosis, benign keratosis, dermatofibroma, vascular lesion, and squamous cell carcinoma^34^. This section will discuss the studies that employed the DL technique to identify SC.

The authors of the article^35^ developed a CAD for categorising 7 subtypes of SC. Pre-processing methods like contrast enhancement, colour space transformation, and brightness adjustment were used to improve the quality of pictures. Subsequently, preprocessed images were used to independently train five CNNs, which included ResNet-50, VGG-16, VGG-19, Xception, and AlexNet. An accuracy of 92.08% was achieved with the ResNet-50 CNN. The study^36^ introduced a DL-based framework for detecting SC lesions. Data augmentation was operated to balance various groups and address data inequality. SC was categorised using InceptionV3, AlexNet, and RegNetY-320. Several combinations of hyperparameters were utilised to optimise the suggested structure. RegNetY-320 achieved the highest performance in F1 score and accuracy, achieving 88.1% and 91%, respectively. Research^37^ developed a dependable method for categorising SC that can distinguish between various types of SC. The system used various image segmentation and classification methods. The combined segmentation paradigm incorporated the threshold approach, edge detection method, region-growing method, clustering method, U-Net model, and RP-Net model. Three CNNs, including ConvNeXtSmall, EfficientNetV2B3, and EfficientNetV2S, were utilised for classification. The accuracy and precision achieved values of 0.984 and 0.986, respectively.

The authors of reference^38^ developed a deep residual neural network (RNN) to detect skin lesions using the wavelet transform. The suggested model utilised the discrete wavelet transform, pooling, and normalisation to eliminate extraneous information from SC photos and uncover more intricate details. Later, the RNN was employed to acquire attributes. Finally, the detection procedure was completed using the Extreme Learning Machine. The F1-Score, specificity, accuracy, precision, and attained values were 93.44%, 98.8%, 95.73%, and 95.84% respectively. The authors of reference^34^ developed a reliable and automated method for detecting seven distinct types of skin cancer by utilising DenseNet and EfficientNet. The highest accuracy of 85.8% was attained by utilising EfficientNet.

The study^39^ utilised various sampling techniques to address the issue of class imbalance. The authors then processed the SC images to eliminate noise. They segmented images using the encoders and decoders approach later on. They used two CNNs including ResNet-50 and DenseNet-169, separately for classification. The study^40^ suggested the use of an effective affine transformation technique for data augmentation to enhance the SC dataset and improve SC identification. SC photos were categorised using Inception-Resnet-v2. After expanding the dataset in this study, the highest reported accuracy achieved was 95.09%. The authors of^8^ suggested an ensemble method that exploited three CNNs, each tailored with unique dropout layers to improve feature-level learning. The combined network named DCENSnet accomplished a superior balance between bias and variance trade-off. The model surpassed the performance of cutting-edge networks on the widely used HAM10000 skin lesion dataset, attaining an accuracy of 99.53%.

In the study^41^, a system was suggested for multiclass SC classification. The CAD had both segmentation and classification steps. ResNet-50-based mask recurrent convolutional neural networks (Mask-R-CNN) and feature pyramid networks (FPN) were implemented during the segmentation phase. A CNN was applied for classification, achieving an accuracy of 86.5%. The study ^42^ processed the SC photographs by applying a decorrelation deformation technique and then used Mask-R-CNN. Important features were acquired and combined from two layers of DenseNet. An SVM classifier was utilised to select the optimal features, resulting in an accuracy of 88.5%. The study^43^ created and evaluated a SC CAD system using DL. Utilising Gated Recurrent Unit Networks (GRU) and an enhanced Orca predation algorithm optimised the effectiveness of the proposed system. The authors also used the HAM10000 skin lesion dataset. On the other hand, the study^44^ introduced an automated framework in which normalisation was applied to dermoscopic images to reduce the effects of noise, outliers, and pixel fluctuations. Malignant areas in the preprocessed images were identified using the Mask-RCNN model. The Mask-RCNN model provided segmentation by precisely outlining the boundary of objects. Discriminatory feature vectors were obtained from the segmented malignant regions using three pretrained CNN models: ResNeXt101, Xception, and InceptionV3. A modified Gated GRU model was used for SC classification, while the study^45^ employed the sand cat swarm optimisation with ResNet50 (SCSO-ResNet50) technique to distinguish deep hidden features from known attributes, providing precise predictions. They use an enhanced harmony search method to optimise attributes and decrease data complexity. Ensemble classifiers such as Naive Bayes, random forest, k-nearest neighbour (k-NN), support vector machine (SVM), and linear regression were employed in the early detection of SC.

## Methods and materials

### HAM10000 Skin cancer dataset

**Figure 1:**
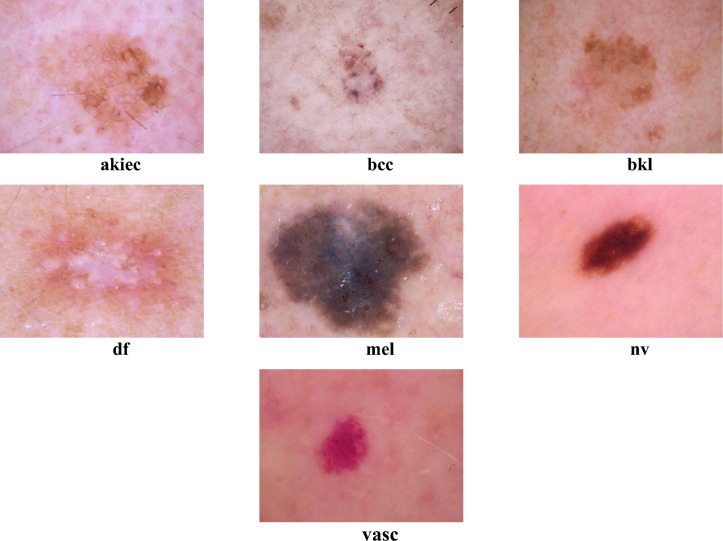
Examples of the HAM10000 dataset.

The HAM10000 data is a benchmark dataset in the fields of SC image analysis and DL research. The dataset includes more than ten thousand dermatoscopic pictures depicting seven distinct forms of SC subcategories. The dataset's value for training and evaluating DL models tasked with critical differential diagnoses lies in its combination of malignant and benign lesions. The images in HAM10000 are carefully labelled by skilled dermatologists, ensuring a reliable reference point for training and assessing models. The dataset shows diversity in patient age, sex, skin type, and lesion location, which promotes the creation of models that can handle real-world variations effectively. The HAM10000 dataset can be accessed for free on the ISIC archive website^46^. The database contains 142 cases of vascular lesions (vasc), 6705 cases of melanocytic nevus (nv), 1113 cases of melanoma (mel), 1099 cases of benign keratosis (bkl), 115 cases of cutaneous fibromas (df), 327 cases of actinic keratosis (akiec), and 514 cases of basal cell carcinoma (bcc). Figure 1 displays numerous images acquired from the HAM10000 database.

### Suggested SCaLiNG CAD

**Figure 2:**
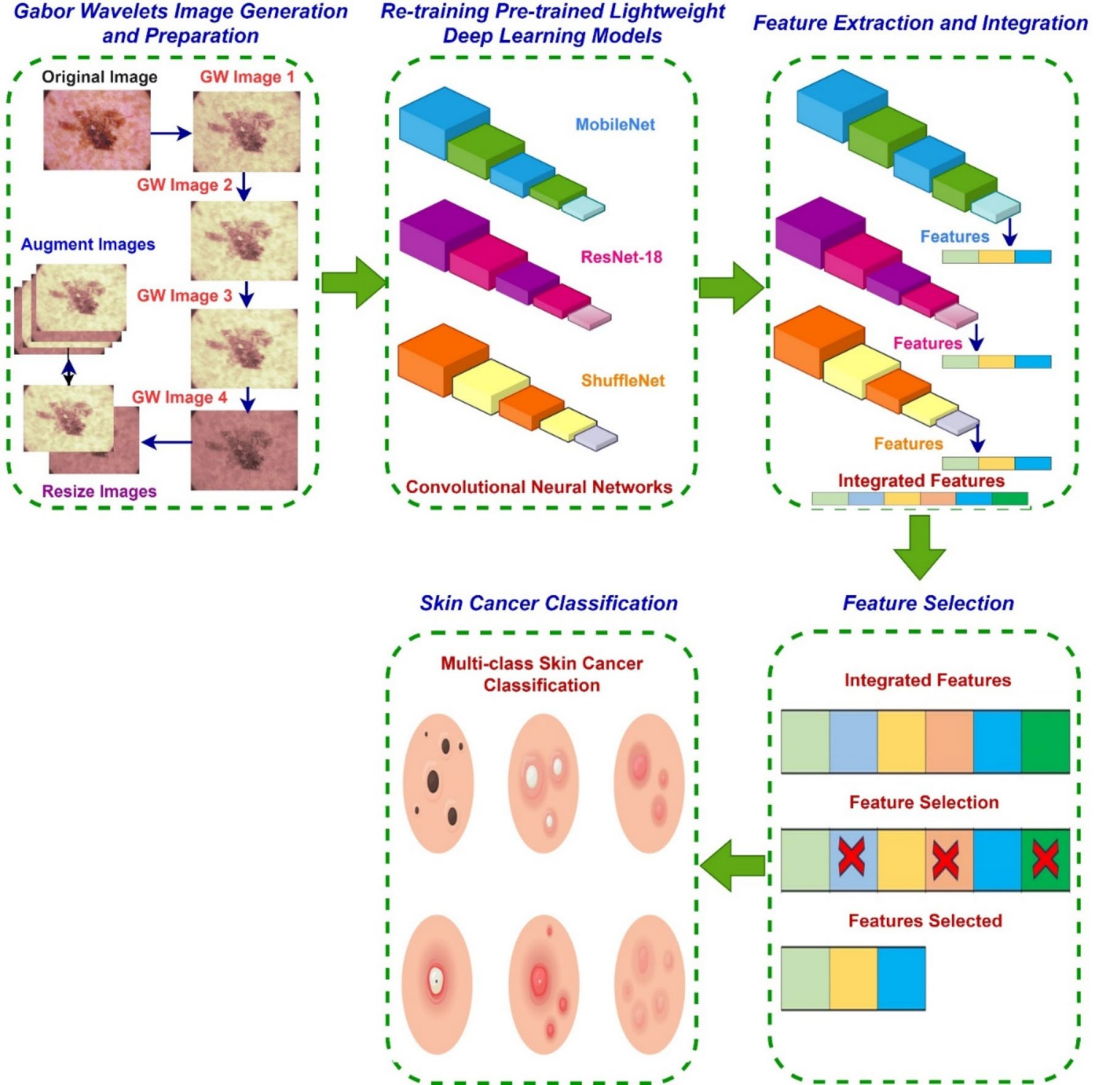
The five stages of SCaLiNG.

The SCaLiNG CAD framework consists of multiple successive phases: GW image generation and preparation, retraining pre-trained lightweight DL models, feature extraction and integration, feature selection, and classification of SC categories. The first step involves the application of GW analysis to dermoscopic pictures. This process generates sub-images that have various orientations and scales, which capture the textural-frequency information. Afterward, both the images that are produced and the original photos are resized and augmented. The previously processed pictures are subsequently inputted into numerous simultaneous versions of pre-trained, compact CNNs of the same architecture. The deep attributes obtained from such CNNs are then combined. This process is repeated for the three CNNs employed in the ScaLiNG tool including ResNet-18, MobileNet, and ShuffleNet. Next, the resultant deep spatial-textural-frequency features, generated by the ResNet-18, ShuffleNet, and MobileNet structures, are merged. Subsequently, a FS procedure is utilised to determine the most distinctive attributes for classification. Ultimately, machine learning classifiers are employed to classify SC by considering the chosen variables. The stages of SCaLiNG CAD are briefly explained in Fig. 2.

#### Gabor wavelets image generation and preparation

The present research leverages GWs to break down SC photos into various frequencies and orientations. GWs produce a collection of transformed photos by identifying important visual characteristics at different sizes and angles. These pictures are to be used to feed pre-trained CNNs to acquire features and classify them. GWs provide a clear benefit compared to conventional image transformation algorithms by simultaneously acquiring textural and frequency domain characteristics. This detailed depiction enables CNNs to acquire a deeper and all-encompassing comprehension of the content within images, which might eventually result in better performance in diagnostic examinations. The GW images are generated using the following formulas:

The GW image is defined as:

1 $$g\left(x,y\right)={e}^{\left[x-{x}_{o}\right]+\left[y-{y}_{o}\right]/{\alpha }^{2}}{e}^{-i\left[{u}_{o}\left(x-{x}_{o}\right)+{v}_{o}(y-{y}_{o)}\right]/{\beta }^{2}}$$

where, the location in a photo is presented as (*x*_*o*_ , *y*_*o*_) and the spatial frequency is referred to as ${\omega }_{o}=\sqrt{{u}_{o}^{2}+{v}_{o}^{2}}$. The orientation is defined as $\theta ={\text{tan}}^{-1}\left(\frac{{v}_{o}}{{u}_{o}}\right)$.

The GW image Fourier transform is given by

2 $$G\left(u,v\right)={e}^{\left[u-{u}_{o}\right]+\left[v-{v}_{o}\right]/{\alpha }^{2}}{e}^{-i\left[{x}_{o}\left({u}_{o}-{v}_{o}\right)+{y}_{o}(v-{v}_{o)}\right]/{\beta }^{2}}$$

The GW function of the real portion is found in the center of the photo *x*_*o*_, *y*_*o*_ = (0,0).

**Figure 3:**
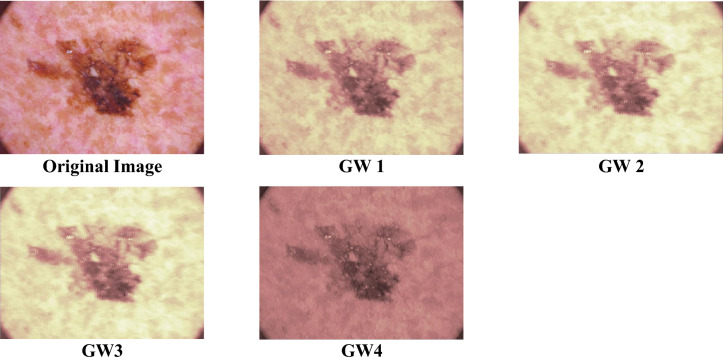
A sample of the four subband images generated after the GW analysis.

GW analysis utilises two scales $x,y$ (0.176, 0.25) and two orientations $\theta$ (0, 90) in this study^33,47^ producing four subband images named: GW 1, GW 2, GW 3, and GW 4. Samples of the images generated after the GW analysis are shown in Fig. 3.

After generating the GW images, both the original and GW-generated photos undergo a resizing process, which involves trimming the lengths of such photographs to align with the input layer length of each CNN. The new dimensions for these images are 224 × 224 × 3 for ResNet-18, ShuffleNet, and MobileNet101. Next, the dataset is divided into sets for training and testing with a proportion of 70%-30%. Subsequently, the data used for training is augmented by applying different augmentation approaches to raise the number of training images, preventing overfitting and improving training performance. Table 1 illustrates the augmentation approaches in detail.

**Table 1:** Augmentation techniques used and their range values.

Augmentation technique	Range
Flipping in horizontal and vertical directions	− 45–45
Scaling	0.5–1.5
Shearing perpendicularly	− 50–50
Rotating in horizontal and vertical directions	− 60–60

#### Retraining pre-trained lightweight DL models

Transfer learning (TL) is used in the current stage to develop and train three efficient pre-trained CNNs that had already been trained on the ImageNet dataset^48^. Specifically, ResNet-18, MobileNet, and ShuffleNet are the CNNs that are being discussed. To match the number of SC categories that are present in the HAM10000 dataset, the total number of fully connected layers of each CNN has been adjusted accordingly to seven. After that, a number of hyperparameters are modified in order to achieve optimal performance; additional information regarding this process can be found in the section titled "Experimental Setting." Following that, the process of retraining the three CNNs begins. While each of the three CNNs is being re-trained independently, individual versions of the created GW images as well as the original images are being used to re-train these CNNs. This indicates that each DL model is trained in parallel with images generated by four GW subbands as well as the original dermoscopic photos.

#### Feature extraction and integration

TL is utilised to extract deep features from the very last fully connected layers of each CNN once the retraining process has been finally completed. The feature vector extracted from each version of a CNN has a length of seven. Immediately after the process of feature extraction comes the procedure of feature integration, which is accomplished in two stages. In the beginning, the deep attributes of each CNN that belong to the four GW images are merged with the deep attributes of the original image. To take advantage of each CNN architecture, the integrated features of the CNNs are then incorporated. In this step. an investigation is carried out to determine whether or not combining deep features from multiple CNNs can improve diagnostic accuracy.

#### Feature selection

FS is an essential method in machine learning that determines the most pertinent features (variables) from a dataset containing a large number of them. The process aims to remove unnecessary and repetitive information, concentrating on features that have a substantial impact on the analysis or model construction. FS is crucial in the medical field due to the diverse range of data, including different measurements, patient histories, and other intricate factors^49,50^. It can be used to enhance model performance, removing irrelevant features allows the model to prioritise the most informative ones, resulting in improved accuracy and generalisability of predictions ^51,52^. FS is also able to decrease computational complexity because having fewer features speeds up the training process of machine learning models and reduces resource usage, which is advantageous for analysing large medical datasets^53^. In addition, FS is capable of improving interpretability by choosing the most significant features that can offer valuable insights into the fundamental biological or clinical connections, assisting medical professionals in comprehending the factors that impact health outcomes.

In this study, the minimum Redundancy Maximum Relevance (mRMR) FS approach is used to select significant features. The mRMR algorithm is a popular method to select features in machine learning tasks. The task at hand involves choosing a subset of features that are not only informative for the target variable, but also have minimal redundancy between them^54^. The mRMR approach accomplishes this by utilising a greedy search method that systematically chooses features with the greatest trade-off between relevance and redundancy. This trade-off can be expressed in various formulations, such as maximising the disparity between relevancy and redundancy or maximising their ratio^55^. The main objective of this study is to ascertain the maximum level of interdependence among a set of attributes *X* and *Y* and the class label *C*, using mutual information (MI) represented as *I*. The MI between two variables can be determined using formula (3) when the marginal probabilities (*p*(*x*) and *p*(*y*)) and the joint probability (*p(x, y)*) for both variables are known^56^.

3 $$I\left(X;Y\right)=\sum_{x\in X}\sum_{y\in Y}p\left(x,y\right)\text{log}\left(\frac{p\left(x,y\right)}{p\left(x\right)p\left(y\right)}\right)$$

Implementing the maximum dependency criterion can be a complex task in multivariate domains. In particular, there is usually a lack of sufficient instances, and calculating the multivariate frequency often requires computationally expensive calculations. A different approach is to determine the criterion that has the greatest degree of significance. The idea of maximum relevance involves the identification of the features that satisfy formula (4). Given an example feature set F = {*f*_*1*_*,f*_*2*_*,…f*_*n*_}

4 $$\text{max}D\left(F,C\right);D=\frac{1}{\left|F\right|}\sum_{{f}_{i}\in F}I\left({f}_{i}, C\right)$$

Selecting variables according to the maximum relevance metric may lead to a considerable amount of duplication. Therefore, it is imperative to incorporate the requirement of minimal redundancy as suggested by the formula (5) mentioned in reference^57^.

5 $$\text{min}R\left(F\right);R=\frac{1}{\left|F\right|}\sum_{{f}_{i}, {f}_{j}\in F}I\left({f}_{i}, {f}_{j}\right)$$

The combination and improvement of the two conditions *D* and *R* lead to the approach of mRMR. Pragmatically, a greedy approach may be employed, where *S* denotes the set of selected features:

6 $${max}_{{X}_{i}\notin S}\left[I\left({X}_{i};C\right)-\frac{1}{\left|S\right|}\sum_{{X}_{j}\in S}I({X}_{i},{X}_{j})\right]$$

#### Skin cancer classification

During the final stage of SCaLinG CAD, skin lesions are classified into one of the seven different categories presented. Specifically, for the purpose of multiclass classification, three SVM classifiers with distinct kernels, namely linear, quadratic, and Gaussian, have been developed, respectively. There are three settings in which the classification procedure is carried out. SVM classifiers are initially trained independently using textural-frequency features obtained from distinct CNNs learned with GW subbands image sets. After that, the SVM classifiers are learned with the aggregated textural-frequency features that are taken from the four GW sub-band images. In the second setting, the spatial features provided by each CNN are combined with the integrated textural frequency features, and the resulting combination is subsequently used as input for the three SVMs. The third setup involves the merging of the combined features of the three CNNs, followed by the utilisation of an FS method to select the most significant features that have an impact on accuracy. A further issue that is investigated in this setting is whether or not the combination of features from multiple CNNs can improve the accuracy of the classification. In addition, it investigates whether or not FS is capable of improving performance.

## Experimental setting

The following section details the hyper-parameter values that have been adjusted and the evaluation metrics used to assess the effectiveness of SCaLinG CAD. Various hyperparameter values have been adjusted for each CNN. Table 2 shows the hyperparameters and the values that correspond to them. All other configurations are kept at their default state. The optimisation method employed to train the CNNs is SGDM, short for stochastic gradient descent with momentum. The codes for the ScaLiNG CAD are available in the supplementary material.

**Table 2:** The CNNs’ hyper-parameters altered and their values.

Hyper-parameter	Value
Mini batch	10
Epochs	40
Learning rate	0.001
Validation frequency	701

Multiple performance metrics are employed for assessing the efficiency of machine learning classifiers. Among them, the accuracy which is a straightforward metric that shows the percentage of correctly classified samples out of the total samples. Precision quantifies the accuracy of positive labels assigned by the model, while recall (or sensitivity) evaluates the accuracy of correctly identified actual positives. Specificity is a metric that quantifies the model's capacity to accurately detect negative cases. It essentially signifies the fraction of actual negative samples that the model correctly identified as negative. The F1-score strikes a balance between precision and recall. The receiving characteristic curve (ROC) and the area under the ROC curve (AUC) are crucial for imbalanced datasets as they evaluate the model's capacity to differentiate between classes at different thresholds. The metrics offer valuable insights into the capabilities and limitations of a machine learning classifier, as well as its suitability for particular uses.

Before calculating performance metrics in machine learning, it is essential to define key indicators: true positive (TP), true negative (TN), false positive (FP), and false negative (FN). TP is the properly determined positive cases, while TN is the accurately categorised negative cases. FP is cases that are mistakenly identified as positive, while FN is cases that are inaccurately classified as negative. The metrics for assessment are computed using the subsequent equations:

7 $$Sensitivity=\frac{TP}{TP+FN}$$

8 $$Specificity=\frac{TN}{TN+FP}$$

9 $$Precision=\frac{TP}{TP+FP}$$

10 $$MCC=\frac{TP\times TN-FP\times FN}{\sqrt{(TP+FP)(TP+FN)(TN+FP)(TN+FN)}}$$

11 $$F1-Score=\frac{2\times TP}{\left(2\times TP\right)+FP+FN}$$

12 $$Accuracy=\frac{TP+TN}{TN+FP+FN+TP}$$

## Results

The section begins with presenting the results of setting I in Section "Setting I classification results" where SVM classifiers are trained independently using textural-frequency features that are extracted by individual CNNs from sets of textural-frequency GW images. In addition, the results of the SVM classifiers when trained using the combined textural frequency features extracted from the four subband images of the GW are illustrated. Afterward, Section "Setting II classification results" illustrates the results of the second setting where the spatial deep features acquired using each DL model are combined with the integrated textural-frequency features obtained by GW and used as inputs for the three SVMs. Finally, in Section "Setting III classification results" the results of setting III are shown where both combined features of all the three CNNs are further integrated, and the mRMR FS approach is employed to select the most significant features that impact accuracy. This setting also investigates the potential enhancement of classification accuracy by integrating features from multiple CNNs. It also investigates whether FS can improve performance.

### Setting I classification results

The results of the SVM classification models trained with deep features obtained using each CNN architecture fed with individual GW subband images of the four subbands generated after the GW analysis and the combined deep features of the four GW subband images are shown in Tables 3, 4, 5 for ResNet-18, ShuffleNet, and MobileNet deep features, respectively. It can be seen from Table 3 that the Linear SVM kernel trained with deep ResNet-18 features exhibited the lowest accuracy among all GW subbands and the combined subbands except for the GW1 and GW3 subbands. The accuracy varies between 71.9% for the GW3 subband and 76.4% for all GW subbands. Furthermore, the quadratic kernel accuracy varies between 69.3% for the GW1 subband and 78.6% for all GW subbands. Furthermore, the Gaussian kernel verified the uppermost accuracy in all scenarios. Accuracy varies between 75.2% for the GW3 subband and 78.4% for all GW subbands.

**Table 3:** Classification accuracy (%) achieved using deep features obtained using ResNet-18 trained with each individual GW subband images.

Deep features	Linear	Quadratic	Gaussian
GW1 Suband	73.3	69.3	75.5
GW2 Suband	73.3	74.6	75.7
GW3 Suband	71.9	71.3	75.2
GW4 Suband	72.9	71.0	75.9
All GW Subands	76.4	78.6	78.4

**Table 4:** Classification accuracy (%) achieved using deep features obtained using ShuffleNet trained with each individual GW subband images.

Deep features	Linear	Quadratic	Gaussian
GW1 Suband	76.3	77.7	78.9
GW2 Suband	75.6	77.5	78.5
GW3 Suband	72.0	73.0	74.6
GW4 Suband	74.8	75.8	78.6
All GW Subands	80.0	82.2	82.2

**Table 5:** Classification accuracy (%) achieved using deep features obtained using MobileNet trained with each individual GW subband images.

Deep Features	Linear	Quadratic	Gaussian
GW1 Suband	79.2	81.6	81.6
GW2 Suband	79.0	80.0	81.6
GW3 Suband	79.1	81.0	81.5
GW4 Suband	79.3	81.1	81.5
All GW Subands	84.0	84.4	84.7

According to Table 4 which exhibits the results using deep features of ShuffleNet, the SVM classifier with a linear kernel had the lowest accuracy in all scenarios. The accuracy varies from 72.0% for the GW3 subband to 80.0% for all GW subbands. For the quadratic SVM, the accuracy ranges between 73.0% for the GW3 subband and 82.2% for all GW subbands. Furthermore, the Gaussian kernel demonstrates a higher level of accuracy in all scenarios than the linear SVM classifier. The accuracy fluctuates between 74.6% for the GW3 subband and 82.2% for all GW subbands. The accuracy of the Gaussian SVM classifier outperforms the other two classifiers. The accuracy of the Gaussian kernel decreased marginally for the GW3 subband (74.6%) in comparison to the other subbands. Across all three kernels, the accuracy of the combined GW subbands is the highest compared to that of each individual subband. This suggests that using all GW subbands together provided the most valuable features for the SVM classifiers.

In accordance with Table 5 which shows the results using deep features of MobileNet, the SVM classifier with the linear kernel has the least accuracy among all GW subbands and the combined subbands. The accuracy differs from 79.2% for the GW1 subband to 84.0% for all GW subbands. In addition, the quadratic kernel outperforms the linear kernel in all scenarios. The accuracy fluctuates from 80.0% for the GW2 subband to 84.4% for all GW subbands. In addition, the Gaussian kernel demonstrates a superior level of accuracy in all respects. The accuracy diverges from 81.5% for GW3 Subband to 84.7% for all GW subbands.

In summary, these findings of Tables 3, 4, 5 indicate that the combined subbands of all GW obtained the highest accuracy compared to each individual subband for all three kernels for deep features ResNet-18, ShuffleNet, and MobileNet. This implies that utilising all of four GW subbands collectively yielded the most informative attributes for the SVM classification model.

### Setting II classification results

**Figure 4:**
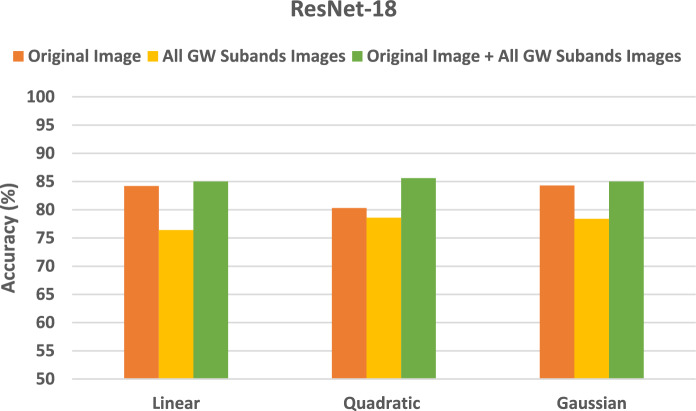
Classification accuracies (%) for the SVM classifiers trained with deep features taken from ResNet-18 CNN trained separately using the original image's deep features, followed by the all-GW subbands' deep features, and lastly the combined deep features of all GW subbands fused with the original image's deep features.

**Figure 5:**
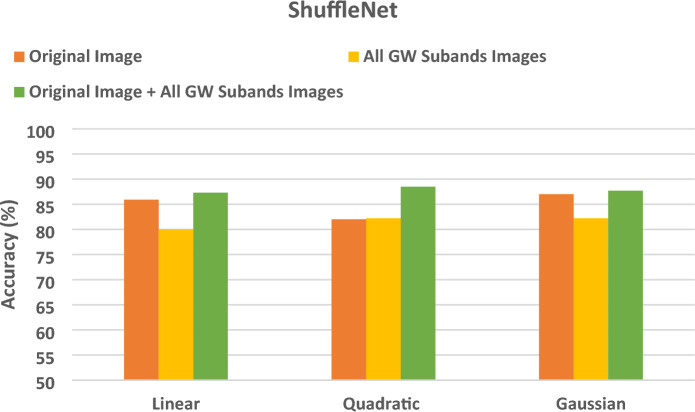
Classification accuracies (%) for the SVM classifiers trained with deep features taken from ShuffleNet CNN trained separately using the original image's deep features, followed by the all-GW subbands' deep features, and lastly the combined deep features of all GW subbands fused with the original image's deep features.

**Figure 6:**
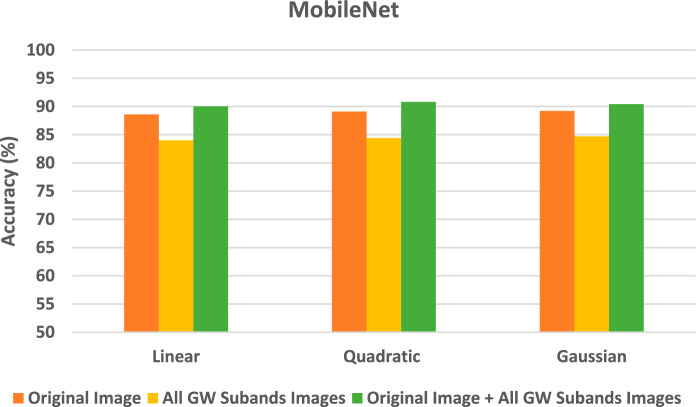
Classification accuracies (%) for the SVM classifiers trained with deep features taken from MobileNet CNN trained separately using the original image's deep features, followed by the all-GW subbands' deep features, an.

The section demonstrates the results of the SVM classifiers when trained with deep features extracted from every DL model learned individually with deep features of the original images, then the deep features of all GW subbands, and finally the deep of both the integrated deep features of all GW subbands fused with deep features of the original images. These results are shown in Figs. 4, 5, 6. Figure 4 reveals that SVM classifiers with different kernels achieved different classification accuracies when trained on deep features extracted from ResNet-18 CNN using various strategies. The Gaussian kernel achieved the highest accuracy in most configurations, except for the original image and the combined GW features, where the quadratic kernel performed better (85.6% and 78.6% for the quadratic compared to 85.0% and 78.4% for the Gaussian kernel, respectively). This indicates that the data displays non-linear relationships, which the non-linear kernels were able to capture more efficiently. Remarkably, the integration of the deep features of the original images with those from all GW subbands yielded a substantial enhancement in the outcomes, surpassing the utilisation of solely the combined GW subbands or the original image alone. Integrating textural-frequency information from GW images with spatial data from the original image could enhance performance.

According to the results in Fig. 5, SVM classifiers have different classification accuracies across various kernels trained on deep features extracted from ShuffleNet CNN. The quadratic and Gaussian kernels consistently outperformed the linear kernel in terms of accuracy across all scenarios. This implies that the data displays non-linear patterns, which the non-linear kernels were able to capture more efficiently. The Gaussian kernel demonstrated superior accuracy in all configurations, except for the integration of four GW subbands combined with the original image (87.7%), where the quadratic kernel (88.5%) exhibited a slightly higher performance. Significantly, the integration of the deep features of the original pictures with those from all GW subbands led to a substantial enhancement in the outcomes, surpassing the utilisation of solely the combined GW subbands or original images alone. This suggests that the combination of spatial and textural-frequency knowledge is more effective than relying solely on spatial data.

The results of Fig. 6 indicate that SVM classifiers with different kernels accomplish varying classification accuracies when learned on deep features obtained from Mobile CNN using different strategies. In all scenarios, the performance of non-linear kernels (quadratic and Gaussian) is superior to that of the Linear kernel. The Gaussian kernel demonstrates superior accuracy across all configurations, except for the original images combined with textural-frequency information of the four GW subbands, where the quadratic kernel exhibited a slightly higher performance (90.8% compared to 90.4%). This indicates that the data displays non-linear relationships, which were better captured by the Gaussian kernel. Outstandingly, when combining the deep features of the original image with those from all GW subbands, the outcomes are enhanced compared to using only the combined GW subbands or the original images. This demonstrates that the integration of spatial and textural-frequency data enhances performance.

d lastly the combined deep features of all GW subbands fused with the original image's deep features.

### Setting III classification results

**Figure 7:**
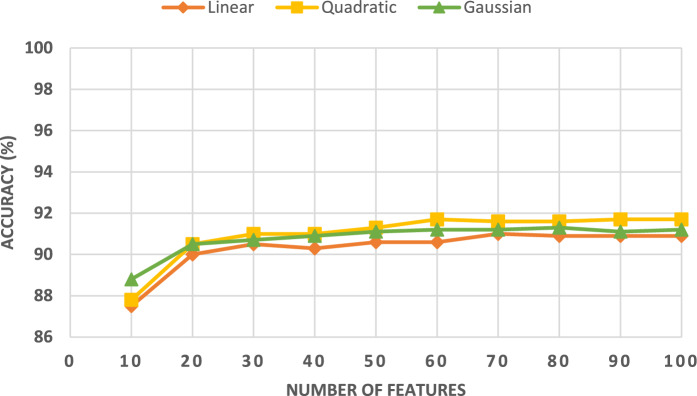
FS classification accuracy (%) of the three SVMs fed with the combined deep features of the three CNNs, including those obtained with the four GW subbands and the original images versus the number of features.

This section illustrates the results of the mRMR FS technique applied to the combined deep features of the three CNNs, including those obtained with the four GW subbands and the original images. An ablation study is shown in Fig. 7 which represents the variation in classification accuracy with increasing number of variables. Figure 7 shows that the classification accuracy improves while increasing the number of features. For the linear kernel, the peak accuracy achieved is 91.0% with only 70 features. Whereas for the quadratic kernel, the maximum accuracy of 91.7% is accomplished with just 60 features. In contrast, the greatest accuracy for the Gaussian kernel is attained with 80 features. These results provide evidence that the mRMR FS is capable of selecting significant features while achieving better performance.

Additional evaluation metrics are employed for evaluating the efficacy of SCaLiNG in achieving the highest level of accuracy after FS for each classifier. The evaluation metrics include precision, specificity, F1-score, MCC, and sensitivity. Table 6 describes these metrics. The linear SVM model achieved a precision of 0.9096, an F1-score of 0.9096, a specificity of 0.9849, an MCC of 0.8946, and a sensitivity of 0.9096. The quadratic SVM classifier yielded a precision of 0.9170, an F1 score of 0.9170, a specificity of 0.9862, an MCC of 0.9032, and a sensitivity of 0.9170. The Gaussian SVM classifier obtained a precision of 0.9126, an F1-score of 0.9126, a specificity of 0.9854, an MCC of 0.8981, and a sensitivity of 0.9126. In general, all three classifiers demonstrated outstanding precision, F1-score, specificity, and sensitivity, with all values exceeding 0.9. This indicates that the mRMR FS process successfully identified a subset of features that provided valuable information for classification purposes.

**Table 6:** Evaluation measures achieved using the MRMR FS approach applied to the combined deep features of the three CNNs including those obtained with the four GW subbands and the original images.

Classifier	Precision	F1-score	Specificity	MCC	Sensitivity
Linear	0.9096	0.9096	0.9849	0.8946	0.9096
Quadratic	0.9170	0.9170	0.9862	0.9032	0.9170
Gaussian	0.9126	0.9126	0.9854	0.8981	0.9126

**Figure 8:**
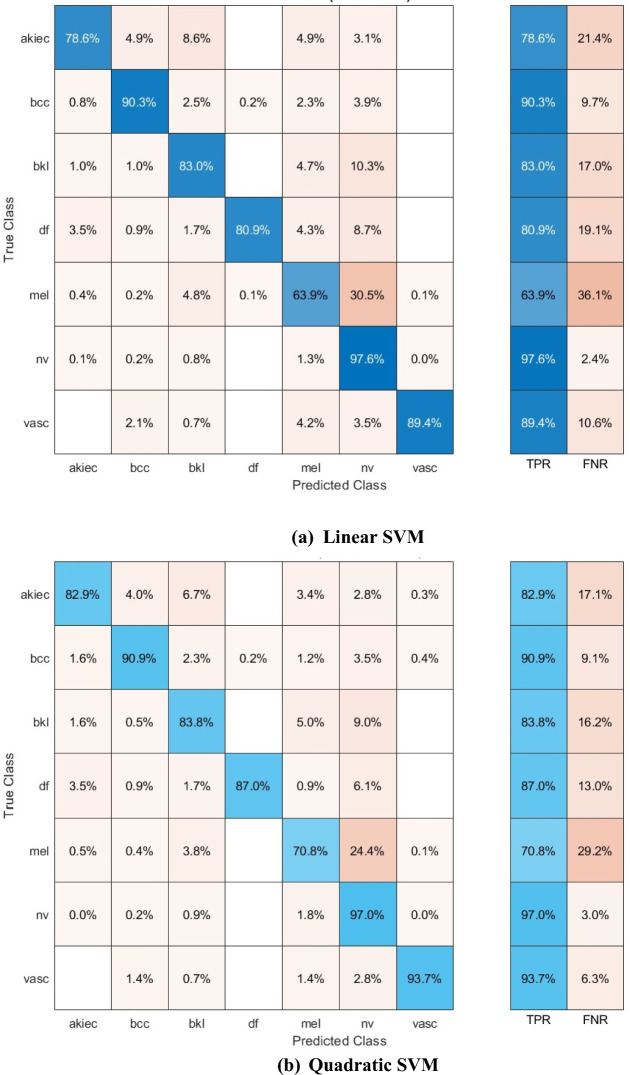

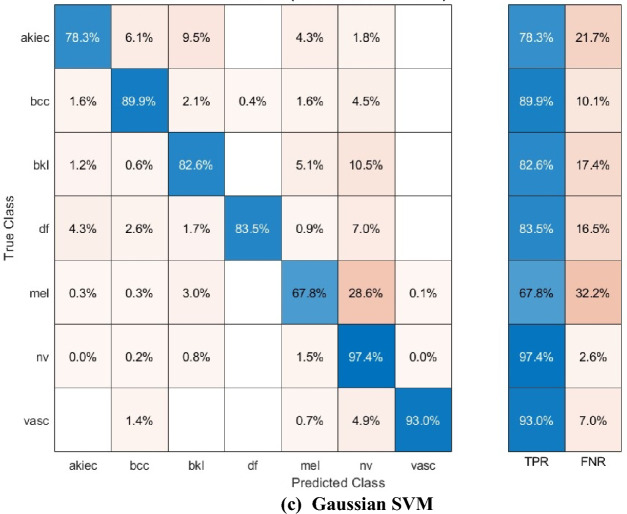
Confusion matrices for the three SVMs fed with the selected features of the mRMR FS approach (**a**) Linear SVM, (**b**) Quadratic SVM, and (**c**) Gaussian SVM.

The confusion matrices for the three SVM classifiers, which were constructed using the selected deep features of the mRMR FS procedure, are displayed in Fig. 8. The diagram demonstrates that the linear, quadratic, and Gaussian SVM models of the SCaLiNG system can accurately classify all categories of the HAM10000 dataset. For the linear, quadratic, and Gaussian SVM classifiers, a sensitivity of (76.6%, 82.9%, 78.3%) for the akiec class, (90.3%,90.9%,89.9%) for the bcc class, (83.0%, 83.8%,82.6%) for the bkl class, (80.9%, 87.0%, 83.5%) for the df class, (63.9%,70.8%, 67.8%) for the mel class, (97.6%, 97.0%,97.4%) for the nv class, and lastly (89.4%, 93.7%,93%) for the vasc class.

**Figure 9:**
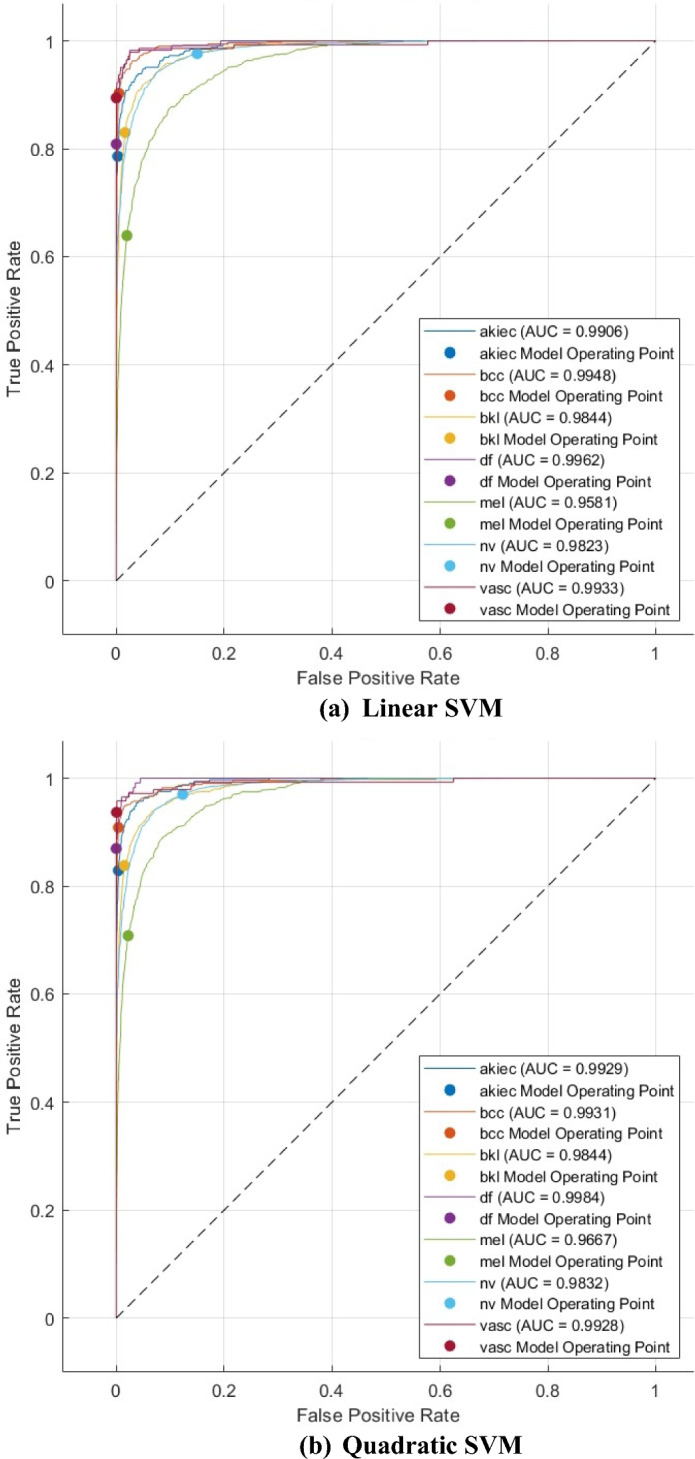

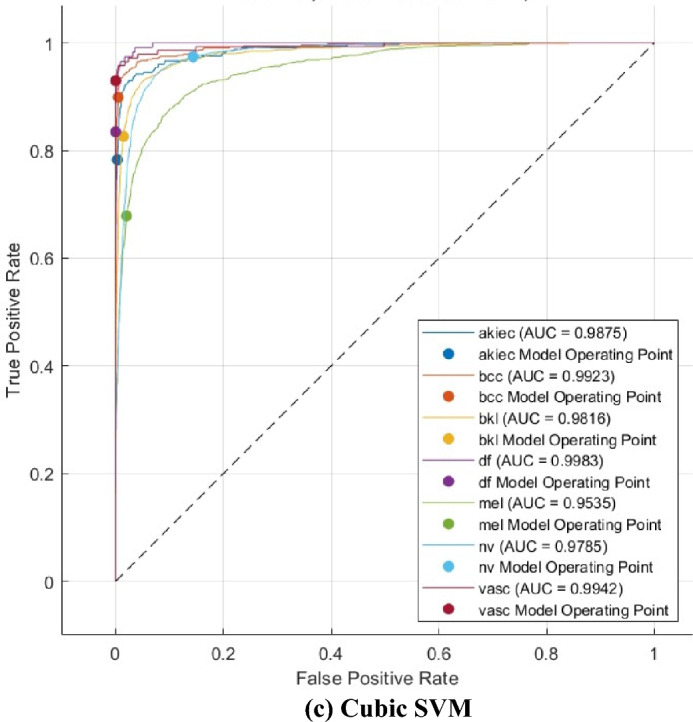
ROC curves for the three SVMs fed with the selected features of the mRMR FS approach (**a**) Linear SVM, (**b**) Quadratic SVM, and (**c**) Gaussian SVM.

The ROC curves for the Linear, Quadratic, and Cubic SVM classifiers are displayed in Fig. 9. These classifiers are applied to the HAM10000 dataset that underwent FS using the mRMR method. The ROC curves plot the true positive rate (sensitivity) on the y-axis and the false positive rate (1-specificity) on the x-axis. Each curve represents a positive class. The SCaLiNG model demonstrates excellent performance across all categories, as evidenced by the ROC curves. All SVM classifiers achieved an AUC value higher than 0.95, which indicates that the SCaLiNG model is capable of accurately distinguishing between various subgroups within SC.

## Discussions

The research article suggests a CAD called "SCaLiNG" for diagnosing SC, which is based on numerous lightweight CNNs and GW. SCaLiNG CAD analyses input images into four directional subbands using a GW-based approach. Each subband provides unique deep features that could be coupled to improve overall performance. Five parallel CNNs of the same topology are used to process the four sub-band images and the original images. Deep features are obtained from every CNN and then merged. Next, those deep features acquired from ResNet-18, ShuffleNet, and MobileNet are integrated. Afterwards, the mRMR FS procedure is implemented to identify the most influential attributes that impact the diagnostic performance. Ultimately, machine learning classification models were used to achieve the task of classification.

**Figure 10:**
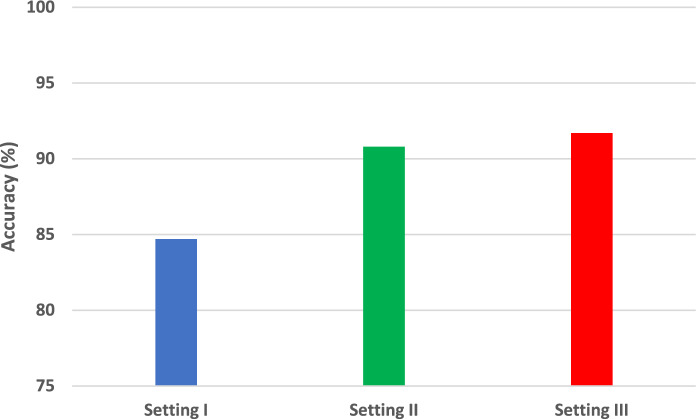
A comparison of the highest levels of accuracy achieved across all SCaLiNG settings.

The procedure for classification is carried out in three distinct settings. At first, SVM classifiers are trained independently using textural-frequency features that are extracted by individual CNNs from sets of GW images. Afterwards, the SVM classifiers are fed with the combined textural-frequency features extracted from the four GW sub-band images. In the subsequent setting, the three SVMs receive input consisting of the combined spatial attributes of every CNN and the integrated textural-frequency features. The third setup combines the features of the three CNNs and employes the mRMR FS method to identify the most critical features that impact accuracy. This setting also investigates the potential enhancement of classification accuracy by combining features from different CNNs. It also examines whether FS has the ability to improve performance. The maximum accuracy achieved in each set-up is demonstrated in Fig. 10.

Figure 10 shows that the integration of both textural–frequency and spatial information (setting II) is superior to relying solely on textual frequency information, (setting I) or spatial information as proven in Figs. 4, 5, 6. This is clear in Fig. 10, since the classification accuracy in setting II (90.8%) is much higher than that reached in setting I (84.7%). Furthermore, the greatest accuracy achieved in setting III is superior to that obtained in settings I and II, which proves that merging deep features from several CNNs of different topologies is capable of enhancing performance. In addition, the increase in performance demonstrates that FS is capable of improving performance while lowering the number of features thus reducing training complexity.

### Comparative evaluation

The outcomes of SCaLiNG CAD are contrasted with comparable studies that developed CAD systems for SC classification utilising the HAM10000 dataset. The superiority of SCaLiNG CAD over various prior CAD systems is demonstrated in Table 7. The accuracy, sensitivity, precision, and F1-score of SCaLiNG are all 0.9170, which is evident to be higher than those observed in previous research, with the exception of the study^42^, which achieved slightly higher accuracy. Although SCaLiNG CAD demonstrated the best overall efficiency, it is worth mentioning that the CAD system^42^ displayed the greatest accuracy of 0.9208. This can be attributed to the use of preprocessing and presegmentation steps, which adds complexity to the model. The exploitation of advanced preprocessing methods and the robust segmentation powers of Mask-R-CNN enable accurate identification of tumour borders, which, in turn, provides useful data for the following feature extraction and classification steps but at the cost of complexity. The reason for the competing ability of SCaLiNG is that it uses both textural-frequency information acquired through the GW method and spatial information extracted from the original images to carry out the diagnosis. In addition, it employs combined deep features from multiple CNNs with diverse architectures. Furthermore, it uses the mRMR FS technique to reduce the dimensions of the feature space, select the most superior features, and eliminate unnecessary attributes.

**Table 7:** Performance comparison of SCaLiNG and other existing CAD systems based on the HAM10000 dataset.

Study	Approaches	Accuracy	Sensitivity	Precision	F1-score
^58^	U-Net based on Efficient Net B4 as a backbone	0.9103	0.8336	0.8444	0.8390
^39^	DenseNet-169	0.9120	–	–	0.9170
^59^	InceptionResNetV2 with Soft-Attention	0.900	0.820	0.810	0.810
^35^	Contrast enhancement, color space transformation illumination correction, ResNet-50	0.8850	–	0.8866	0.8860
^42^	Decorrelation deformation for preprocessing. Mask-R-CNN for segmentation LS-SVM for feature selection and classification	0.9208	0.9253	0.9373	0.9274
^36^	RegNet-320	0.9100	–	–	0.8810
^60^	Haar hair removal + EVGG	0.8900	0.8900	0.8900	0.8800
^61^	20-layer CNN for segmentation + 17 layered CNN for classification + Regula Falsi + Extreme learning machine (ELM)	0.8702	0.8698	–	–
SCaLiNG	GW + MobileNet + ShuffleNet + ResNet-18 + mRMR + Quadratic SVM	0.9170	0.9170	0.9170	0.9170

### Skin-CAD limitations and forthcoming directions

It is necessary to investigate the limitations of the suggested SCaLiNG CAD for SC diagnosis. One main issue revolves around the possible influence of an unequal distribution of classes in the training dataset. The lack of balance in the data could result in a shortage of training data for certain subclasses of SC, which may undermine the effectiveness of the classification process. The presence of class imbalance may have a substantial impact on the effectiveness of machine learning algorithms, possibly resulting in predictions that are skewed toward the majority class. Within the framework of SCaLiNG, this may result in an excessive focus on categorising the most forms of skin cancer with a larger number of samples, while not performing as well in identifying subtypes. with a lower number of images In order to address the aforementioned issue, multiple techniques are implemented throughout the SCaLiNG system. Initially, data augmentation approaches are employed to artificially increase the number of images. Furthermore, employing numerous CNN architectures in an ensemble manner may alleviate the effects of class imbalance by offering varied feature representations and possibly increasing the resilience of the model. In addition, the inclusion of feature selection is intended to determine the most distinguishing attributes, which can assist in mitigating the impact of imbalanced class variations on the classification procedure. Furthermore, the effectiveness of the SCaLiNG CAD is examined by employing measures such as precision, recall, and F1-score, in order to offer a more thorough evaluation of its efficacy on datasets with imbalanced data. Although the above steps are put in place to tackle the issue of class imbalance, ScaLiNG shows varying performance across different SC classes. For instance, SCaLiNG exhibits higher performance for nv, bcc, and vasc in comparison to other SC categories. Therefore, it would be beneficial to conduct additional research on advanced methods, including cost-sensitive learning or class weighting, to improve the system's effectiveness on imbalanced datasets. Future research will investigate other sampling techniques to address class imbalance issues.

Though this study specifically examined the identification of different types of SC, the basic concepts and techniques of SCaLiNG could potentially be applied to various other skin disorders. The ScaLiNG system's ability to efficiently extract and merge spatial-textural-frequency features by integrating compact CNNs and GW is a flexible strategy that can be applied to various tasks involving the evaluation of dermatological images. In order to assess the model's capability to be applied to a wider range of skin disorders, future investigations might include utilising SCaLiNG on datasets that cover a more diverse array of skin conditions. Furthermore, the ScaLiNG model's adaptability might be enhanced further by considering the integration of various other imaging techniques, including dermoscopy and clinical photography.

It is important to clarify that although the SCaLiNG CAD exhibits promising efficacy in categorising subtypes of SC, the main objective of the present study is to construct and assess a reliable classification model, without specifically taking into account individual patient features. Therefore, the model's ability to accurately recognise patients with specific characteristics including age, gender, or skin form, is not directly evaluated. This stated shortcoming can be addressed in future research by adding patient-specific data into the model, which has the possibility of improving its effectiveness and practicality. Furthermore, it would be worthwhile to investigate the establishment of submodels customised for particular patient populations.

ScaLiNG lacks a pre-segmentation stage. The choice to skip this stage is mainly driven by the aim of simplifying the procedure and decreasing the intricacy of computing. The goal behind ScaLiNG is to utilise the inherent capability of CNN networks to determine relevant characteristics without depending on intentional segmentation by simply entering the whole dermoscopic picture into the suggested CAD. Although removing pre-segmentation makes the CAD workflow less complex, it is recognised that this may create difficulties. For example, ScaLiNG could be required to acquire the ability to differentiate between the tumor and surrounding areas without explicit instruction. This has the potential to affect the precision of the system, particularly when the tumour only occupies a tiny area of the photo. To address this issue, the SCaLiNG CAD tool integrates numerous CNNs that operate across distinct image resolutions and scales. This approach enables the models to efficiently gather attributes at varying degrees of detail. Moreover, the utilisation of Gabor wavelets offers further texture data that may help identify and describe lesions.

The integration of any CAD framework, such as SCaLiNG, into an actual clinical environment presents numerous challenges. One significant obstacle is the attainment of generalisability. The performance of the SCaLiNG framework, which is developed on a particular set of images, may not be consistent across various patient groups that have various colours of skin, tumor forms, or imaging techniques used with various scanning devices. To address this issue, it is necessary to conduct external validation experiments that involve collecting data from a broader and more varied sample of patients from different scan centres. The wider range of data enables the model to effectively generalise and adjust to practical clinical situations. Moreover, it is essential to acquire trust and approval from medical professionals. To incorporate the SCaLiNG tool into the current clinical process, it is essential to engage together with dermatologists in order to comprehend their requirements and apprehensions. This cooperation could assist in customising the design and capabilities of the system to seamlessly integrate into their diagnostic procedure. Furthermore, thorough clinical assessments conducted by qualified dermatologists are imperative to evaluate the efficacy in a real-life context and verify its dependability in aiding the diagnosis of skin cancer. To overcome such obstacles, SCaLiNG CAD could be strategically placed to gain greater acceptance and enhance its clinical effectiveness for a more diverse patient population.

In order to improve the dependability and suitability of the CAD in a clinical environment for a broader range of individuals, it is essential to collaborate with specialists and conduct external validation which will be done in future work. There are plans to collect data from a larger group of patients from different scan centers to enhance the generalisability and performance of the study In the end, the system will be evaluated by professional dermatologists in a clinical environment.AD will be examined in clinical settings by experienced dermatologists.

## Conclusion

Although CNN-based CAD systems have been developed to assist dermatologists, these tools have certain limitations. Presently, many CAD systems depend on complex CNN structures, which may impede their widespread use. In addition, they often rely on an individual CNN model, which restricts the ability to capture a wide range of image features. Additionally, such CADs mainly focus on analysing the spatial information of the original images, while disregarding potentially valuable directional or textural details. This paper introduced a CAD called ‘SCaLiNG’ to identify and categorising SC at an early stage using a combination of three efficient CNNs of distinct structures (ResNet-18, ShuffleNet and MobileNet) and GW. SCaLiNG preserved four directional details by utilising GW to decompose the input photos into four directional subbands producing textural-frequency information. Five simultaneous CNNs of the same architecture were fed with the original image that produced spatial information and the four sub-bands that provided multiple textural-frequency representations. By merging the extracted deep spatial-textural-frequency features of each CNN architecture, a more comprehensive representation and enhanced classification accuracy were achieved. Additionally, SCaLiNG merged deep spatial-textural-frequency features of the three CNNs to combine the benefits of each unique structure, thus improving performance. It also used mRMR FS to choose the influential features. SCaLiNG CAD showcased its efficacy in the classification of multi-class skin lesions, attaining noteworthy outcomes. The proposed model attained an accuracy of 0.9170 using just 60 features. SCaLiNG CAD exhibited a superior enhancement in overall accuracy when compared with other existing CAD systems. These findings indicate that SCaLiNG is a reliable and effective CAD model for the detection, classification, and analysis of cancerous skin lesions. The results significantly enhance the progress of current knowledge in the early detection of skin cancer.

## Supplementary Information

Supplementary Information 1.

Supplementary Information 2.

## Data Availability

The HAM10000 dataset analysed during the current study are available in the Harvard Dataverse repository, https://dataverse.harvard.edu/dataset.xhtml?persistentId=doi:10.7910/DVN/DBW86T (accessed 30 May 2023).
